# Immunogenicity and Efficacy of a Trivalent HSV-2 gC2, gD2, gE2 Nucleoside-Modified mRNA-LNP Vaccine Against HSV-1 Eye Infection and Neuroinvasion in Mice

**DOI:** 10.3390/vaccines14030253

**Published:** 2026-03-10

**Authors:** Alyssa Chalmin Katz, Kevin P. Egan, Zauraiz Syeda, Sarah Son, Bahiyah Watson, Manaswini Gopalakrishnan, Valerie Bromberg, Enrico Radaelli, Charles-Antoine Assenmacher, Sita Awasthi, Gary H. Cohen, Harvey M. Friedman

**Affiliations:** 1 University Laboratory Animal Resources, University of Pennsylvania, Philadelphia, PA 19104, USA; achalmin@vet.upenn.edu; 2 Department of Pathobiology, School of Veterinary Medicine, University of Pennsylvania, Philadelphia, PA 19104, USA; enrada@vet.upenn.edu (E.R.); chasse@vet.upenn.edu (C.-A.A.); 3 Infectious Disease Division, Perelman School of Medicine, University of Pennsylvania, Philadelphia, PA 19104, USA; kevinpe@pennmedicine.upenn.edu (K.P.E.); zauraizs@gmail.com (Z.S.); sarah.son1@pennmedicine.upenn.edu (S.S.); bahiyah.watson@pennmedicine.upenn.edu (B.W.); manaswini.gopalakrishnan@pennmedicine.upenn.edu (M.G.); valbromberg11@gmail.com (V.B.); sawasthi@pennmedicine.upenn.edu (S.A.); 4 Basic and Translational Sciences, School of Dental Medicine, University of Pennsylvania, Philadelphia, PA 19104, USA; ghc@upenn.edu

**Keywords:** HSV-1, HSV-2, eye infection, neuroinvasion, blepharoconjunctivitis, trivalent mRNA vaccine, genital herpes, serum neutralizing antibodies, glycoproteins gC2, gD2, gE2

## Abstract

Background/Objectives: Eye infection with herpes simplex virus type 1 (HSV-1) can result in keratitis, a leading cause of corneal blindness. We evaluated whether an experimental vaccine containing HSV-2 immunogens to prevent genital herpes also protects against HSV-1 eye infection and neuroinvasion. Methods: Mice were immunized twice, one month apart, with PBS or a nucleoside-modified lipid nanoparticle vaccine containing mRNA encoding for gC2, gD2, and gE2. One month later, 10^6^ plaque forming units (PFU) (10 lethal dose 50, LD_50_) of the HSV-1 McKrae strain were added to the intact cornea of each eye. Results: The vaccine prevented death and markedly reduced eyelid and attached conjunctival inflammation (blepharoconjunctivitis) and weight loss compared with the PBS group. Tissues from the ocular conjunctiva and eye bulb, olfactory bulb/peduncle, trigeminal ganglia, and brain (brainstem, cerebrum, and cerebellum) were harvested 5 days post-infection from 5 mice each in the PBS and vaccine groups, and from another 10 mice in the vaccine group 7 weeks post-infection. At 5 days, HSV-1 was not detected in any tissue in the vaccine group, while viral titers were positive in 16 of 25 (64%), and HSV-1 DNA was detected in 22 of 25 (88%) individual tissues in the PBS group. Histopathological and immunohistochemical analysis at 5 days post-infection confirmed that the vaccine protected against inflammation; however, some animals experienced breakthrough blepharoconjunctivitis. At 7 weeks, 3 of 10 (30%) mice in the vaccine group had HSV-1 DNA detected in the eyes or trigeminal ganglia tissues, but no animal had HSV-1 DNA detected in brain tissues. The vaccine produced cross-reactive HSV-1 neutralizing antibodies and gD1 IgG binding antibodies, but low or undetectable cross-reactive binding antibodies to gC1 and gE1. Conclusions: Despite occasional mild, localized breakthrough infections, the vaccine provided disease-modifying immunity and was neuroprotective. The results suggest that a single herpes vaccine effective against genital HSV-2 may be neuroprotective against HSV-1 following eye infection.

## 1. Introduction

*Herpesviridae* is a family of DNA viruses that cause both symptomatic and latent infection. The ability to establish a latent infection poses unique challenges for investigating treatments and cures, as viruses in latent reservoirs, such as neural or lymphoid tissues, are challenging to target, leading to recurrent infections that are more common and aggressive in immunosuppressed individuals [1]. Herpes simplex virus type 1 (HSV-1) causes oral herpes and is highly prevalent, affecting 67% of the global population [2,3]. Herpes simplex virus type 2 (HSV-2) is the primary cause of genital herpes and is less common, affecting 13% of the global population [4,5]. No FDA-approved vaccines are available to prevent or treat HSV-1 or HSV-2, resulting in unmet needs.

Several HSV envelope glycoproteins are involved in virus entry and immune evasion from antibodies and complement [6,7,8]. Three of these glycoproteins are included as immunogens in an experimental HSV-2 genital herpes vaccine: gC2, gD2, and gE2 [9]. These immunogens were selected to prevent virus entry mediated by gD2, to prevent gC2 from inhibiting complement activation, and to prevent gE2 from blocking antibody-mediated activities via the IgG Fc domain. Preclinical studies assessing the efficacy of the HSV-2 *gC2/gD2/gE2* vaccine against HSV-1 oral infection and HSV-1 and HSV-2 genital lesions, as well as HSV-2 neonatal herpes, have revealed disease-modifying immunity, manifest by a marked reduction in both lesions and latent infection [9,10,11].

HSV-1 eye infection is the most common infectious agent worldwide to cause corneal damage (keratitis) [12,13,14,15]. After primary eye infection, HSV-1 establishes latency in the trigeminal ganglia and reactivates periodically, leading to recurrent corneal infections, irreversible corneal opacity, neovascularization, and blindness [16]. Additionally, HSV-1 eye infection can lead to viral trafficking to the brain, resulting in encephalitis [17,18]. Reactivation of HSV-1 infection and its associated inflammatory responses have received considerable attention in recent years due to their potential contribution to neurodegenerative conditions such as Alzheimer’s Disease [19,20,21,22,23,24,25].

Mouse models of primary ocular infection often scarify the cornea to facilitate virus infection [26,27,28]. The McKrae strain of HSV-1 is a more neuroinvasive strain that causes ocular infection and encephalitis without the need for scarification and has been used in vaccine challenge studies [29,30]. Infecting eyes on intact corneas without corneal scratching results in lower mortality, which is beneficial for pathogenesis studies and is consistent with the natural pathogenesis of HSV-1 ocular infection in humans [31].

An effective vaccine to prevent and treat herpes simplex virus is urgently needed for public health. Our vaccine candidate encodes three HSV-2 glycoproteins; however, due to similarities between HSV-1 and HSV-2 glycoproteins, the vaccine exhibits cross-reactivity and prevents HSV-1 infection [10,32]. Previous studies investigating the efficacy and immunogenicity of the trivalent HSV-2 mRNA vaccine against HSV-1 orolabial infection revealed reduced clinical disease and decreased viral DNA in the trigeminal ganglion when compared with a PBS control [10]. The current study aims to determine the efficacy and immunogenicity of the trivalent HSV-2 *gC2*, *gD2*, *gE2* nucleoside-modified mRNA-lipid nanoparticle vaccine against primary HSV-1 eye infection in mice.

## 2. Materials and Methods

### 2.1. Ethical Statement

Female BALB/c mice (Charles River Laboratories) were 6–8 weeks old when they arrived in our mouse colony. The University of Pennsylvania Institutional Animal Care and Use Committee approved the animal studies reported here under protocol 805187.

### 2.2. Cell Lines and Virus

Vero cells (ATCC, ref CCL-81) were used for all cell culture procedures. The McKrae strain of HSV-1 was generously provided by Homayon Ghiasi [29].

### 2.3. Immunization of Mice

Mice were immunized intramuscularly in a hind limb with 10 μg mRNA (3.33 μg each of g*C2*, *gD2*, and *gE2*) encapsulated in lipid nanoparticles and administered in a volume of 30 μL [9]. Mice were immunized twice, 28 days apart, with the HSV-2 trivalent mRNA-LNP vaccine or with PBS.

### 2.4. HSV-1 Eye Infection

Mice were challenged with HSV-1 McKrae strains at doses ranging from 10^3^ to 10^6^ plaque forming units (PFU). Viral challenge occurred 30 days after the second immunization. Mice were briefly anesthetized with a solution of 100 mg/kg ketamine and 12.5 mg/kg xylazine. The virus was administered in a volume of 5 µL, applied to the intact cornea of each eye using a micropipette. Before the animals were returned to their cages, the virus was allowed to rest on the surface of the eye for 5 min, undisturbed, during which much of the inoculum pooled in the conjunctival sac.

### 2.5. Monitoring and Humane Endpoints

Animals were monitored daily for 14 days post-infection for survival, weight loss, blepharoconjunctivitis, and corneal clouding as an indicator of keratitis. Blepharoconjunctivitis is defined as inflammation of the eyelids, characterized by swelling, redness, and/or crusting, while red eyes characterize conjunctivitis. Mice received between 0 and 4 points for blepharoconjunctivitis per eye. A score of 0 indicated no observable signs of blepharitis or conjunctivitis. A score of 1 indicated minimal eyelid swelling or redness, a score of 2 indicated moderate eyelid swelling and/or crusty discharge, a score of 3 indicated severe eyelid swelling with moderate periocular alopecia or skin lesions, and a score of 4 indicated severe eyelid swelling with the eye completely shut and/or severe periocular alopecia or skin lesions. Humane endpoints included a score of 3 or greater in at least one eye, a weight loss of ≥20%, or systemic signs of illness, such as lethargy, hunching, or dehydration.

### 2.6. Tissue Collection and Storage

Eye tissues collected included the eye bulb and ocular conjunctiva, but not eyelids. The neuronal tissues included olfactory bulbs, trigeminal ganglia, brainstem, cerebrum, and cerebellum. Tissues were collected either at 5 days or 7 weeks post-infection. They were then stored individually in 400–500 μL of DMEM supplemented with 5% FBS at −80 °C until processed.

### 2.7. Tissue Virus Titers

Tissues were subjected to 3 cycles of freezing and thawing before measuring virus titers. We used a glass grinder to homogenize eyes, and a motorized pestle to homogenize all other tissues. Serial 10-fold dilutions of homogenates were evaluated by plaque assay on Vero cells. 200 µL of the homogenate was placed on the cells, and the final calculated titer was adjusted to account for the homogenate volume.

### 2.8. Serum IgG ELISA Titers and Neutralizing Antibody Titers

Serum was collected via the submandibular vein 1 month after the second immunization, just before infection. Sera were stored at −80 °C until evaluated for IgG binding antibodies by ELISA and for neutralizing antibodies. IgG ELISA endpoint titers against gC1, gD1, and gE1, or gC2, gD2, and gE2 were performed as previously described [9,10]. Neutralizing titers were evaluated using a plaque reduction assay, where 2-fold serial dilutions of mouse sera were incubated with 100 PFU of the HSV-1 McKrae strain at 37 °C for 1 h. Five percent human serum from an HSV-1/HSV-2 double seronegative donor was used as a source of complement for neutralizing antibody assays. The neutralizing titer was defined as the highest dilution that reduced plaques by ≥50% [10].

### 2.9. HSV-1 DNA Copy Number in Tissues by qPCR

DNA was purified using the QIAcube HT benchtop system (Qiagen, Germantown, MD, USA). 5 µL of each sample was combined with 20 µL of master mix containing HSV-1 US9 primers (Integrated DNA Technologies, Coralville, IA, USA Forward: 5′ACG GCC TCG CCA GTT TC3′, Reverse: 5′TTG GCC GCC TCG TCT TC3′) and probe (Applied Biosystems, Waltham, MA, USA 6FAM TCG AAG CCT ACT ACT CG MGBNFQ) or adipsin primers (Integrated DNA Technologies, Forward: 5′GCA GTC GAA GGT GTG GTT ACG3′, Reverse: 5′GGT ATA GAC GCC CGG CTT TT3′) and probe (Applied Biosystems, 6FAM CGA GGC CGC CAA C MGBNFQ), and TaqMan (Applied Biosystems, 4352042). Samples were plated in duplicate wells and amplified for 40 cycles using the LightCycler 96 (Roche Diagnostics, Indianapolis, IN, USA). Samples were considered negative if both wells contained fewer than 1 HSV-1 DNA copy. Samples were rerun in triplicate if only one of the duplicate wells was positive. The sample was considered positive if at least two of the triplicates were positive. To determine HSV-1 DNA copy number, we ran purified HSV-1 DNA (ATCC, VR-539DQ) or adipsin DNA (BioChain, Princeton, NJ, USA, D1334149) as standards. These standards, ranging from 1 copy to 5 × 10^4^ DNA copies, were plotted to generate a standard curve, which was used to calculate the HSV-1 DNA copies in the samples. This curve must have a slope of −3.3 with a 10% variance allowance, an R-squared value close to 1, and an efficiency value close to 2. The limit of quantification (LoQ) value for each plate is defined as 10 times the standard deviation of the standards divided by the slope of the standard curve. The HSV-1 DNA concentration was then divided by the adipsin DNA concentration of the same sample and multiplied by 1 × 10^5^ to determine the copies of HSV-1 DNA per 10^5^ adipsin genes. The Log_10_ of this value was graphed [10,32].

### 2.10. Histopathological and Immunohistochemical Analysis

A subset of animals was used to evaluate lesions by histopathology (H&E) and immunohistochemistry (IHC). For histopathology, the entire head, including the eyelids, conjunctiva, eye bulbs, nasal and oral cavities, and the brain, was fixed in 10% neutral buffered formalin for 2–3 days, and was decalcified in 15% formic acid overnight. Coronal sections were obtained at the level of the nasal cavities and the eyes. Sagittal sections of the brain included the nasal peduncles, the trigeminal ganglia, the brainstem, the cerebrum, and the cerebellum. Samples were routinely processed for hematoxylin and eosin (H&E) staining and assessed for pathological changes, including inflammation, cellular or tissue damage, and viral intranuclear inclusion bodies.

For IHC, 5 μm-thick slices of the tissues used for histopathological evaluation were mounted on ProbeOn™ slides (Thermo Fisher Scientific, Waltham, MA, USA) and stained using the Leica BOND RXm automated platform and the Bond Polymer Refine Detection kit (Leica, Teaneck, NJ, USA, DS9800). Epitope retrieval was performed with the BOND ER2 high pH buffer (Leica #AR9640) for 20 min at 98 °C. Endogenous peroxidase was inactivated with 3% H_2_O_2_ for 10 min at room temperature (RT). The Leica PowerVision IHC/ISH Super Blocking solution (PV6122) was applied for 30 min at RT to block nonspecific tissue-antibody interactions. A rabbit polyclonal primary antibody against HSV-1 (Abcam, Waltham, MA, USA, ab9533) at a concentration of 1:1000 was diluted with the same blocking solution and incubated on the slides for 45 min at RT. An HRP-conjugated anti-rabbit IgG was then applied for 25 min at RT and served as an IHC detection system. Immunoreactivity was revealed with the diaminobenzidine (DAB) chromogen reaction. Slides were then routinely counterstained in hematoxylin and a cover slip applied with a resinous mounting medium (Thermo Scientific ClearVue™ coverslip). Samples from infected animals served as positive controls. Negative controls were obtained by either omitting the primary antibody or replacing it with an irrelevant isotype-matched rabbit polyclonal antibody. The slides were then assessed for the presence and distribution of a positive signal.

### 2.11. Statistics

Prism version 10.6.1 for macOS was used for statistical analysis. The statistical tests used to derive the *p* values are listed in the figure legends. *p* < 0.05 was considered significant.

## 3. Results

### 3.1. Lethal Dose 50 (LD_50_) Determination and Scoring for Disease Severity

**Figure 1:**
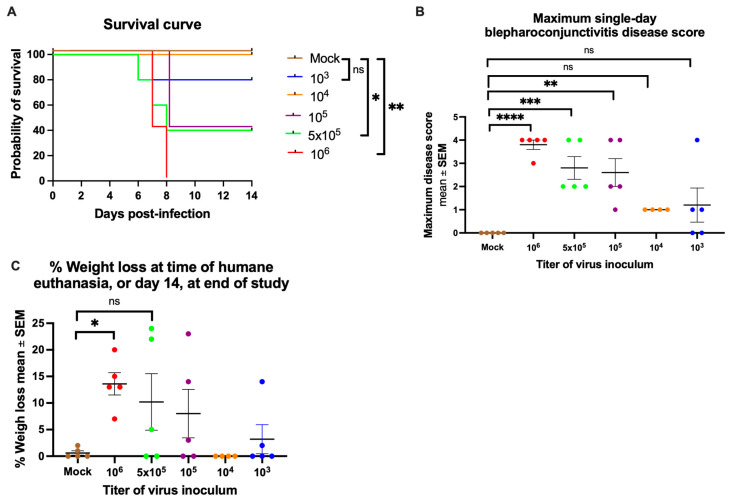
Inoculation of HSV-1 produces dose-dependent changes in survival, blepharoconjunctivitis, and weight loss. HSV-1 was inoculated onto the intact cornea of each eye. (**A**) Survival curve. *p* values calculated by the Log-rank test. *n* = 5 mice/group (**B**) The blepharoconjunctivitis disease score is shown for the most affected eye on the most severe day of disease. *n* = 5 mice/group. (**C**) Percent weight loss at time of humane euthanasia or at the end of the study on day 14 post-infection. *n* = 5 mice/group, except *n* = 4 for the 10^4^ group. *p* values for (**B**,**C**) were calculated by Ordinary one-way ANOVA and Dunnett’s multiple comparisons test. * *p* < 0.05, ** *p* < 0.01, *** *p* < 0.001; **** *p* < 0.0001; ns, not significant.

Mice were mock-infected using uninfected cell lysates or infected with purified HSV-1 McKrae strain at 10^6^, 5 × 10^5^, 10^5^, 10^4^, or 10^3^ PFU that was pipetted onto the intact cornea of each eye. All 5 mice inoculated with 10^6^ PFU reached a humane endpoint between days 6 and 8 post-infection. Mice inoculated with 5 × 10^5^ or 10^5^ PFU each had 3 of 5 animals reach a humane endpoint between days 6–8 post-infection, while animals inoculated with 10^4^ or 10^3^ PFU had 0 of 4 or 1 of 5 animals succumb to infection, respectively (Figure 1A). The calculated LD_50_, which is the lethal dose for 50% of animals, was 10^5^ PFU, calculated based on an online program (https://probitanalysis.wordpress.com/) that required entering the proportion of animals at each inoculation titer that lived or required humane euthanasia.

We plotted the severity of blepharoconjunctivitis (inflammation of the eyelids and conjunctivae) based on the highest severity score each animal achieved, ranging from 0 to 4 as described in Materials and Methods (Figure 1B). The mean disease severity for each group decreased with declining virus titers used to infect the eyes, except for one animal in the 10^3^ group, which developed severe blepharoconjunctivitis. Weight loss followed a pattern very similar to that of blepharoconjunctivitis (Figure 1C), with the highest doses resulting in the greatest weight loss. No corneal opacity or neurologic disease was observed. We conclude that applying the highly virulent HSV-1 McKrae strain to the cornea without scarification produced reliable, dose-dependent disease, providing a model for assessing the efficacy of the trivalent mRNA vaccine for preventing HSV-1 eye infection.

### 3.2. Kinetics of Infection After Virus Inoculation

**Figure 2:**
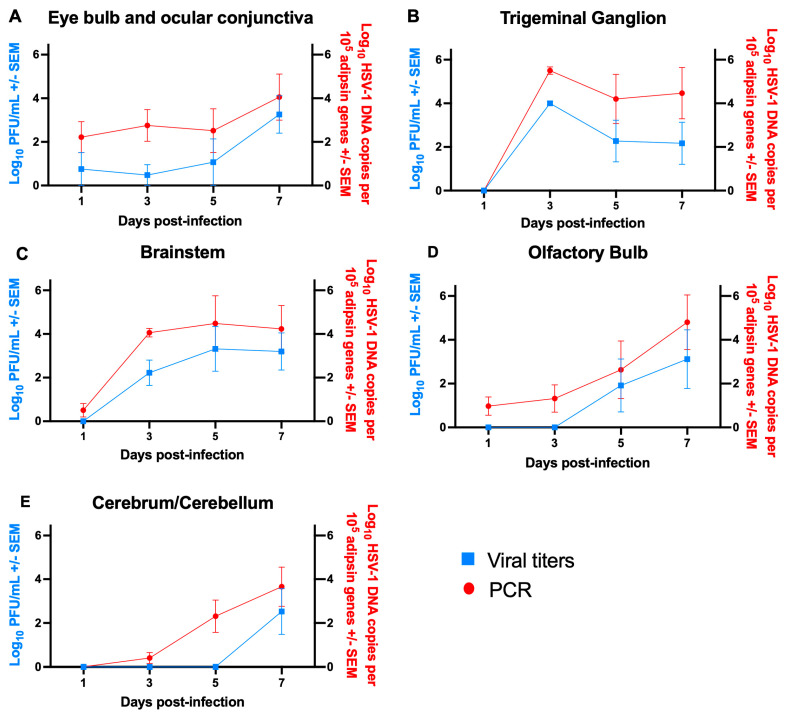
Kinetics of infection after HSV-1 was placed on the intact cornea of each eye. Animals were infected with 10^5^ PFU (1 lethal dose 50, LD_50_) of HSV-1 in each eye and sacrificed on days 1, 3, 5, and 7 post-infection (*n* = 5 per group). Each tissue was processed separately and analyzed for virus titers (blue) and HSV-1 DNA copy number by qPCR (red). The tissues included (**A**) eye (eye bulb and attached ocular conjunctiva), (**B**) trigeminal ganglion, (**C**) brainstem, (**D**) olfactory bulb, and (**E**) cerebrum/cerebellum.

Animals were infected with HSV-1 using 10^5^ PFU (1 LD_50_) McKrae strain applied to each intact cornea. Animals were sacrificed on days 1, 3, 5, and 7 post-infection to investigate the virus’s movement from the eye to the brain. The eye bulbs and attached ocular conjunctivae, olfactory bulbs, trigeminal ganglia, brainstems, and cerebrums/cerebellums were collected and analyzed for viral titers and HSV-1 DNA, expressed as DNA copy number per 10^5^ adipsin genes. On day 1 post-infection, the virus was present in the eye bulb and ocular conjunctiva at low viral titers (<1 log_10_) but was not detected in other tissues (Figure 2A, blue curve). By day 3, the virus was recovered from the trigeminal ganglia and brainstem, on day 5 from the olfactory bulb, and on day 7 from the cerebrum/cerebellum (Figure 2B–E, blue curves). HSV-1 DNA copy number in tissues followed a similar pattern, except evidence of infection was detected earlier by HSV-1 DNA qPCR than by virus culture (Figure 2A–E, red curves). We conclude that the virus spreads from the eye to the trigeminal ganglia and brainstem, and then to the cerebrum/cerebellum. Virus spread to the olfactory bulb lags behind that to the trigeminal ganglia and brainstem, likely because the virus reaches the nose and olfactory bulb via the lacrimal duct, which delays its appearance in the nose after eye inoculation [26].

### 3.3. Antibody Responses

**Figure 3:**
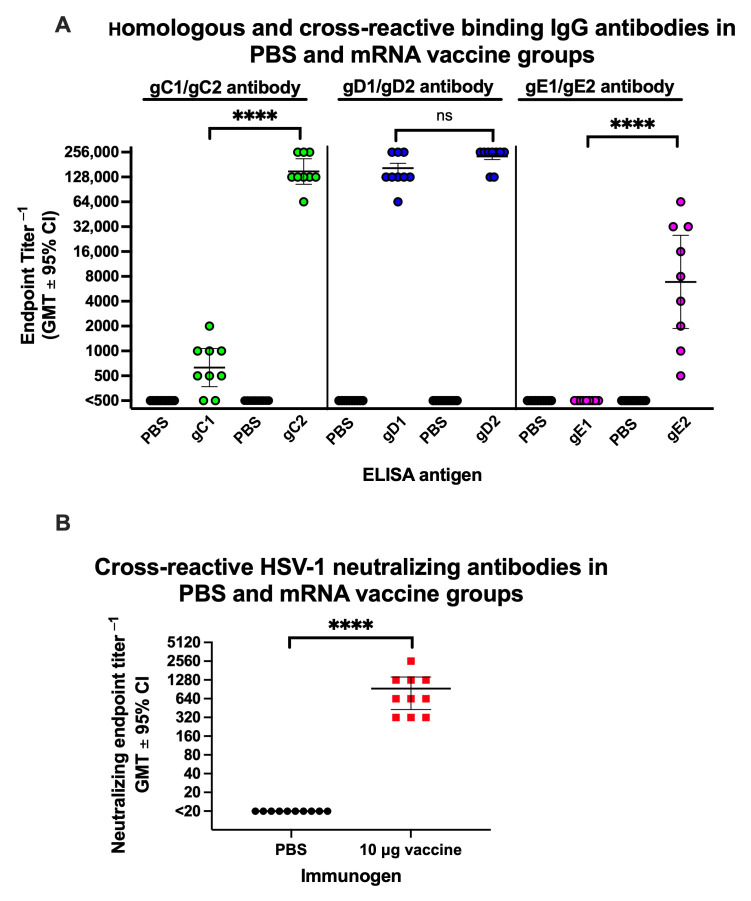
Serum IgG ELISA (binding) and neutralizing antibodies. Mice were immunized twice IM with PBS or 10 μg of the trivalent HSV-2 mRNA vaccine. Sera were obtained 4 weeks after the second immunization, before infection with HSV-1. (**A**) Serum IgG ELISA endpoint titers against HSV-2 or cross-reacting antibodies against HSV-1. (**B**) Neutralizing antibodies to HSV-1. *n* = 9 or 10 animals/group. *p* values were calculated using the two-tailed Mann–Whitney test. **** *p* < 0.0001; ns, not significant.

Animals were immunized with PBS or 10 μg of the trivalent HSV-2 mRNA-LNP vaccine. Binding IgG titers were measured by ELISA using sera obtained just before infection and 1 month after the second immunization. The sera were evaluated for antibody titers to HSV-1 gC1, gD1, and gE1, as well as HSV-2 gC2, gD2, and gE2. In the PBS group, IgG ELISA titers were <1:500 for each glycoprotein (Figure 3A). We detected cross-reactive antibody titers of 1:750 to gC1, which were significantly lower than the gC2 titers of 1:160,000 (Figure 3A, left panel). The cross-reactive gD1 titer of 1:160,000 did not differ significantly from the gD2 titer of 1:225,000 (Figure 3A, middle panel). We detected no cross-reactive gE1 antibodies, while the gE2 titers were 1:18,000 (Figure 3A, right panel). These IgG ELISA results are similar to those previously reported by our laboratory and indicate that antibodies produced against gD2 are mainly type-common. In contrast, antibodies against gC2 and gE2 are mostly type-specific [10].

Serum neutralizing antibody titers were performed on the same sera in the presence of 5% human complement. Mice immunized with the mRNA vaccine produced cross-reactive HSV-1 geometric neutralizing antibody titers of 1:920 (Figure 3B). We conclude that the trivalent HSV-2 mRNA vaccine produces cross-reactive antibodies that neutralize HSV-1, presumably because antibodies to gD2 are the main contributors to neutralization, and these antibodies are type common.

### 3.4. Vaccine Challenge Studies

**Figure 4:**
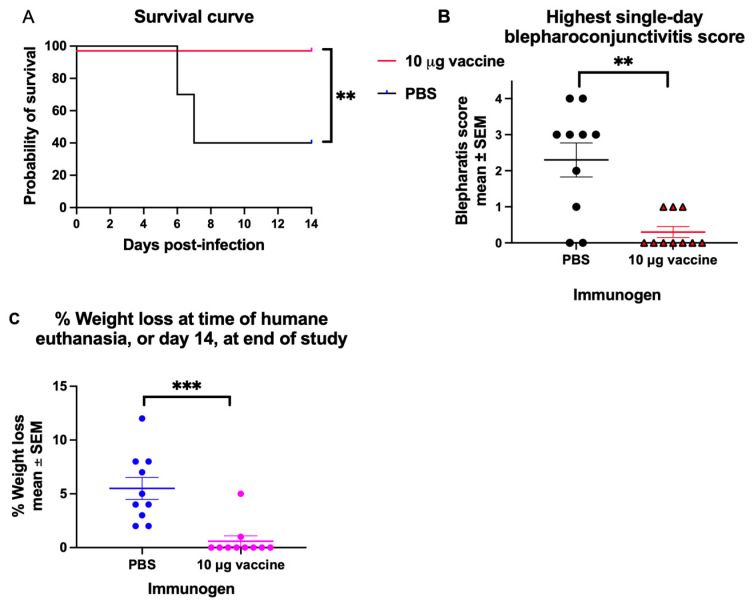
Vaccine protection against disease after HSV-1 inoculation on the intact cornea of each eye. Mice were immunized with PBS or 10 μg trivalent mRNA, and 28 days after the second immunization, the intact cornea of each eye was inoculated with 10^6^ PFU (10 LD_50_) HSV-1 (*n* = 10/group). (**A**) Survival curve. The *p* value was determined using the Log-rank test. (**B**) Blepharoconjunctivitis disease score on the most severe day of disease post-infection for each animal. (**C**) Weight loss. *p* values for (**B**,**C**) were calculated using the two-tailed Mann–Whitney test. *n* = 10/group; ** *p* < 0.01; *** *p* < 0.001.

To determine whether the vaccine protects against HSV-1 eye infection, animals were immunized twice, on days 0 and 28, with PBS or 10 μg of the trivalent *gC2/gD2/gE2* mRNA. Four weeks later, animals were infected with 10^6^ PFU (10 LD_50_) of HSV-1 McKrae placed on the intact cornea of each eye. All 10 animals that received the trivalent mRNA vaccine survived to the end of the experiment on day 14, compared with 4 of 10 animals that received PBS (Figure 4A). We scored the animals for blepharoconjunctivitis. We plotted the peak disease day for the eye with the most severe blepharoconjunctivitis for each animal. 8 of 10 mice in the PBS group developed blepharoconjunctivitis, with severity ranging from 1 to 4. In contrast, 3 of 10 animals in the vaccine group developed blepharoconjunctivitis, each with a severity score of 1 (Figure 4B). The mean peak blepharoconjunctivitis score in the mock group was significantly higher at 2.5 compared to 0.3 in the vaccinated group (Figure 4B). All animals in the PBS group lost weight. In contrast, 8 of 10 mice in the vaccine group maintained their weight post-infection, while one animal had 5% weight loss and another had 1% weight loss (Figure 4C). We conclude that immunization with 10 μg of the trivalent HSV-2 mRNA-LNP provided disease-modifying immunity, as evidenced by total protection against death and near-total protection against blepharoconjunctivitis and weight loss.

### 3.5. Protection by Immunization Against Tissue Infection

**Figure 5:**
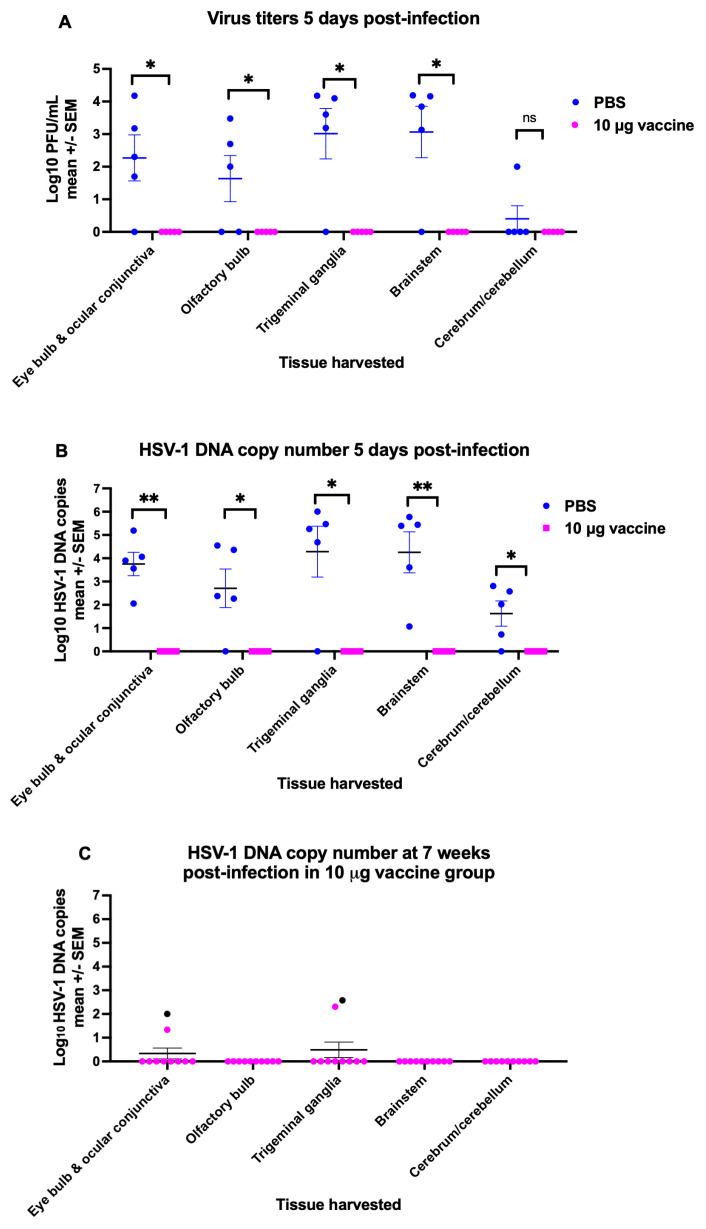
Vaccine protection as assessed by virus culture and HSV-1 DNA copy number in tissues after HSV-1 inoculation of the intact cornea of each eye. Mice were immunized twice with PBS or 10 μg of the trivalent HSV-2 mRNA vaccine, and challenged in each eye with 10^6^ plaque forming units (PFU) HSV-1 (10 LD_50_). (**A**) Tissues were harvested 5 days post-infection for viral titers in PBS and vaccine groups (*n* = 5/group). (**B**) The same tissues were evaluated for HSV-1 DNA copy number. (**C**) Tissues were harvested 7 weeks post-infection for HSV-1 DNA copy number in mice in the vaccine group (*n* = 10). The black symbol indicates the same mouse with HSV-1 DNA in the eye and the trigeminal ganglia. *p* values were calculated by the two-tailed Mann–Whitney test; * *p* < 0.05; ** *p* < 0.01; ns, not significant.

Animals were immunized twice with PBS or 10 μg of the trivalent mRNA vaccine, and 4 weeks later, 10^6^ PFU (10 LD_50_) of HSV-1 was applied to the intact cornea of each eye. Five days post-infection, five mice each from the PBS group and the mRNA vaccine group were euthanized, and tissue samples from the eyes (eye bulbs and attached ocular conjunctivae), olfactory bulbs, trigeminal ganglia, brainstems, cerebrums/cerebellums were harvested for virus titers and HSV-1 DNA copy number. Four of 5 animals in the PBS group had virus recovered from the eyes, trigeminal ganglia, and brainstems, while 3 of 5 had virus recovered from the olfactory bulbs, and 1 of 5 had virus recovered from the cerebrum/cerebellum. In contrast, no virus was recovered from any tissue of vaccinated animals (Figure 5A). HSV-1 DNA was detected in all tissues evaluated from the 5 animals in the PBS group, except for 1 animal that was negative for HSV-1 DNA from cerebrum/cerebellar tissues, while no animal in the vaccine group had HSV-1 DNA detected in any tissue (Figure 5B). We conclude that at 5 days post-infection, the HSV-2 trivalent mRNA vaccine was efficacious in protecting the eye bulb, the attached ocular conjunctiva, and neuronal tissues (neuroprotective immunity), including the olfactory bulb, trigeminal ganglia, brainstem, and cerebrum/cerebellum.

We next evaluated tissues for evidence of HSV-1 DNA persistence at 7 weeks post-infection in 10 animals in the vaccine group. Two of 10 animals had HSV-1 DNA detected in samples from the eyes and trigeminal ganglia; one of these mice had HSV-1 DNA detected in both tissues (Figure 5C, indicated by a black symbol), while two other mice had HSV-1 DNA detected either in the eye or the trigeminal ganglia. In total, 3 mice had HSV-1 DNA detected in the eyes or the trigeminal ganglia. Olfactory bulb, brainstem, and cerebrum/cerebellum tissues were negative for HSV-1 DNA in all 10 mice (Figure 5C). We conclude that a few animals had breakthrough infection of the eye and the trigeminal ganglia. Still, no animal had a breakthrough infection of the brainstem, cerebrum, or cerebellum.

### 3.6. Histopathology and Immunohistochemistry

**Figure 6:**
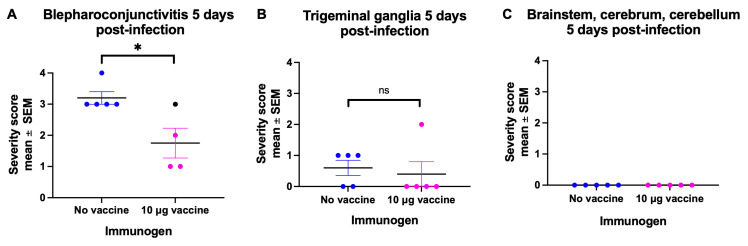
Infection severity based on histopathology and immunohistochemistry. Five days post-infection, (**A**) Blepharoconjunctivitis (eyelids, conjunctivae), (**B**) trigeminal ganglia, and (**C**) brains (brainstem, cerebrum, and cerebellum) were processed for H&E and IHC and evaluated for disease severity. Animals were either not immunized (no vaccine) or immunized with 10 µg of the mRNA vaccine (vaccine), with 4 or 5 tissues evaluated per animal. The black symbol in the vaccine group identifies the animal with a single IHC-positive cell. *p* values were calculated by the two-tailed Mann–Whitney test; * *p* < 0.05, ns, not significant.

Five mice that were not vaccinated and 5 mice that were immunized with 10 μg of the mRNA vaccine were euthanized 5 days post-infection. The eyelids, palpebral and ocular conjunctivae, eyeballs, including the corneas, trigeminal ganglia, brainstems, cerebrums, and cerebellums were harvested for histopathological and immunohistochemical analysis. Note that the eye tissues obtained for histopathology and immunohistochemistry differ from those obtained for virus cultures and HSV-1 DNA in that the histopathology assessment included the eyelids and palpebral conjunctivae, which were not included in the viral studies. The H&E and IHC slides of these tissues were evaluated by Veterinary Pathologists (C-AA, ER). Both Pathologists scored the H&E and IHC slides, and both were blinded to vaccine status. As a marker of disease severity, we scored the blepharoconjunctivitis on a scale of 0 to 4, where 0 represented no inflammation, 1 represented minimal inflammation with scattered inflammatory cells in the eyelid dermis and conjunctival substantia propria, 2 represented mild inflammation with low numbers of inflammatory cell infiltrates, 3 represented moderate inflammation with a denser inflammatory cell infiltrate, and 4 represented severe inflammation with high numbers of inflammatory cells and frequent tissue damage in the overlying haired skin and/or conjunctival mucosa. The inflammatory responses consisted of neutrophilic and lymphoplasmacytic infiltrates, as well as HSV intranuclear inclusions. Intranuclear inclusions were detected in one or more tissues of each unimmunized animal but in none of the immunized animals. All 5 unimmunized animals had moderate or severe blepharoconjunctivitis. In contrast, the 4 immunized animals (tissue was not available for one) had either minimal, mild, or moderate inflammation (Figure 6A). The animal with moderate inflammation in the vaccine group had a single conjunctival epithelial cell that stained positively for HSV-1 antigens by IHC (Figure 6A, black symbol). This same animal had mild inflammation in the trigeminal ganglia (Figure 6B). The single IHC-positive cell in the vaccine group animal and the positive results for HSV-1 DNA in the eyes and trigeminal ganglia from 3 of 10 animals at 7 weeks (Figure 5C) suggest that the blepharoconjunctivitis and trigeminal ganglia inflammation were caused by breakthrough infection rather than immune-mediated disease. No animals in either group at day 5 showed corneal inflammation, possibly because we did not use corneal scarification to infect the cornea. No animals in either group showed inflammation in the brainstem, cerebrum, or cerebellum, perhaps because the inflammatory response lags behind the virus’s arrival in these tissues (Figure 6C). We conclude that the H&E and IHC results support the viral studies (Figure 5A–C).

**Figure 7:**
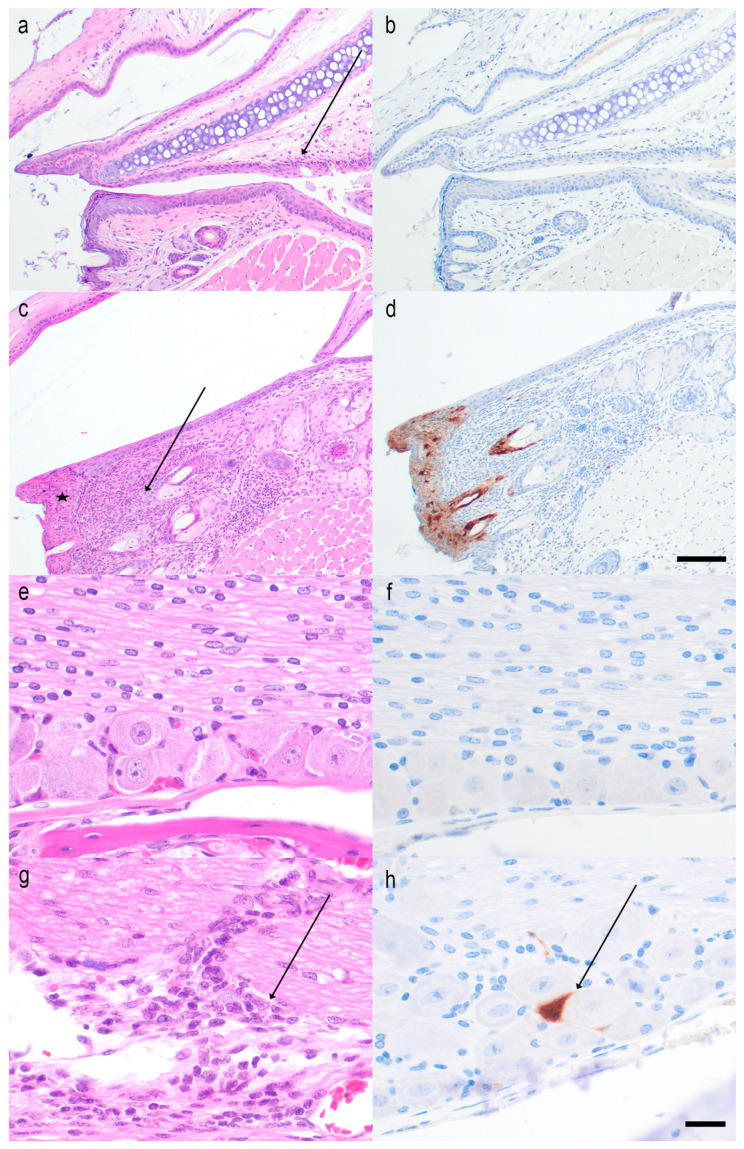
Representative images of the eyelids and palpebral conjunctivae, and the trigeminal ganglia of mice infected with HSV-1 that were unvaccinated or vaccinated with the trivalent HSV-2 mRNA vaccine. (**a**,**b**) Evaluation for blepharoconjunctivitis in an immunized animal: The eyelid shows minimal mixed inflammatory cell infiltrates in the conjunctival substantia propria (arrow). No HSV-1 immunolabelling is detected by IHC. H&E (**a**) and IHC for HSV-1 (**b**). (**c**,**d**) Evaluation for blepharoconjunctivitis in an unimmunized mouse: Blepharoconjunctivitis is visible (arrow) with regional necrosis of the epithelium (asterisk) and strong immunolabeling of the surface and follicular epithelium for HSV-1. H&E (**c**) and IHC for HSV1 (**d**). Scale bar for (**a**–**d**), 100 µm. (**e**,**f**) Trigeminal ganglion of an immunized mouse: The trigeminal ganglion shows no significant findings. H&E (**e**) and IHC for HSV-1 (**f**). (**g**,**h**) Trigeminal ganglion of an unimmunized mouse: Meningitis is present in the meninges surrounding the trigeminal ganglion (arrow), and occasional neurons exhibit strong immunolabeling for HSV-1. H&E (**g**) and IHC for HSV1 (**h**). Scale bar for (**e**–**h**), 20 µm.

Representative photomicrographs of the H&E and IHC stains are shown of the eyelids, conjunctiva, and trigeminal ganglia of 1 unimmunized mouse, and of two representative mice in the vaccine group (Figure 7). The photomicrographs support the clinical observations of extensive disease in unimmunized mice and mild breakthrough infection in some immunized mice.

## 4. Discussion

We used the mouse eye infection model to evaluate whether an experimental trivalent *gC2*, *gD2*, *gE2* mRNA-LNP vaccine, designed to prevent HSV-2 genital infection, also protects against HSV-1 eye infection (blepharoconjunctivitis) and neuroinvasion when the virus is applied to the intact cornea. In unimmunized mice, HSV-1 spread from the eye to the trigeminal ganglia and brain as reported by others [33]. The HSV-2 vaccine did not confer sterilizing immunity, as evidenced by clinical observations of blepharoconjunctivitis, which were supported by H&E and IHC studies. Additional evidence for non-sterilizing immunity was that 1 of 5 immunized animals had inflammation in the trigeminal ganglia at 5 days post-infection, and HSV-1 DNA was detected in the trigeminal ganglia of 3 of 10 animals at 7 weeks post-infection. Notably, the HSV-2 vaccine provided complete protection of the brain, as evident by negative viral cultures, HSV-1 DNA assays, H&E, and IHC of brainstem, cerebrum, and cerebellum at 5 days post-infection, and HSV-1 DNA assays at 7 weeks post-infection, suggesting the vaccine prevents neuroinvasion. Other investigators have reported that an HSV-1 *gC*, *gD*, and *gE* vaccine, administered either as an mRNA or an adjuvanted subunit protein vaccine, was neuroprotective against intranasal inoculation with HSV-1 McKrae in mice. However, the extent of protection was less than reported here [34]. We conclude that the HSV-2 *gC2*, *gD2*, *gE2* mRNA vaccine provided incomplete cross-protection against localized HSV-1 eye infection at the inoculation site but strong cross-protection against neuroinvasion. Without corneal scarification, we did not detect keratitis in vaccinated or unvaccinated mice; therefore, we cannot comment on the vaccine’s corneal-protective effect.

Analysis of immune responses to the HSV-2 vaccine provides insights into potential areas for improvement. We demonstrated that the HSV-2 trivalent vaccine produced high titers of cross-reactive neutralizing antibodies to HSV-1 and IgG binding antibodies (ELISA) to gD1. However, we detected much lower cross-reactive binding antibodies to gC1 and no measurable cross-reactive binding antibodies to gE1. We previously reported that the HSV-2 vaccine elicited robust CD8^+^ T cell responses to gE1-overlapping peptides [10]. These T cell studies were performed using the same dose of the mRNA vaccine as in the current study and were not repeated here. Future studies can evaluate cytokine responses of CD4^+^ and CD8+ T cells and tissue-resident memory T cells in eyelid and trigeminal ganglia tissues after HSV-1 challenge, and clarify the contributions of neutralizing antibodies, antibody-dependent cellular cytotoxicity, and T cell responses to vaccine-induced protection. These additional studies will help define the modifications required to improve the protection provided by the HSV-2 trivalent vaccine.

A prior study in humans reported that a gD2 subunit vaccine protected against HSV-1 infection. However, it did not protect against HSV-2, indicating that type-common immune responses to gD2 may be sufficient to prevent HSV-1 infection [35]. Our results suggest that we can improve upon the excellent disease-modifying protection provided by the HSV-2 mRNA vaccine by incorporating additional immunogens. Nevertheless, the HSV-2 vaccine prevented neuroinvasion, an important observation given the life-threatening consequences of HSV-1 encephalitis [36]. Limitations of the study include evaluating only female BALB/c mice and the HSV-1 strain McKrae. Expanding the scope of the studies to include males, other neuroinvasive HSV-1 strains, such as H129, and eye infections in guinea pigs and rabbits would provide valuable information [37,38,39,40,41].

Efforts to develop vaccines for preventing or treating genital herpes in people have been fraught with difficulty. Two subunit protein vaccine candidates for preventing genital herpes, gD2 and gB2/gD2, failed to achieve their primary endpoints [35,42,43]. Multiple other vaccine candidates, including live virus, DNA, nucleoside-modified mRNA, and subunit protein vaccines, entered human trials but were discontinued (NCT04222985; NCT02030301; NCT02837575; NCT06033261; NCT03146403; NCT01687595; NCT00231049, NCT06033261). Only one mRNA vaccine candidate is currently in human trials in the USA (ClinicalTrials.gov NCT05432583). The focus of all these vaccine trials was on genital herpes rather than oral herpes, which may be driven by market considerations, including greater commercial interest in vaccines for genital herpes than for oral herpes. Two events may shift thinking, not away from genital herpes as a vaccine target, but towards a pan-herpes simplex vaccine that is effective against both genital and oral herpes. These events include emerging evidence that the inflammatory response triggered by recurrent HSV-1 and HSV-2 infections may increase the risk of Alzheimer’s disease, as well as studies such as the one reported here, suggesting that a herpes vaccine may be effective against both HSV-1 and HSV-2 [10,23,24,25,32]. If an effective HSV-2 vaccine is approved for genital herpes, epidemiological studies may help establish a link between HSV-1 and/or HSV-2 infection and neurodegenerative diseases, as noted for the shingles vaccine [44].

Independent of a potential link to neurodegenerative diseases, HSV-1 keratitis and encephalitis are serious complications of HSV-1 infection. Once latency is established in the trigeminal ganglion, regardless of the initial route of infection, reactivation is possible, leading to viral transport to the cornea or brain and ensuing keratitis or encephalitis [45,46]. The ability of the trivalent HSV-2 vaccine to protect against trigeminal ganglion infection, as shown in this study, could reduce the incidence of keratitis, a sight-threatening infection, and encephalitis, a life-threatening illness, while also lowering the incidence of genital herpes. The results reported here are encouraging for the development of a pan-herpes simplex vaccine.

## 5. Conclusions

An experimental vaccine designed to prevent genital herpes caused by HSV-2 provided cross-protection against HSV-1 eye infection and neuroinvasion when a virulent HSV-1 strain, McKrae, was applied to the intact corneas of mice immunized with a nucleoside-modified mRNA lipid nanoparticle vaccine expressing gC2, gD2, and gE2. The vaccine provided disease-modifying immunity, as no animals in the vaccine group required humane euthanasia, and only a few developed localized disease (blepharoconjunctivitis) at the inoculation site or had weight loss. The vaccine was highly neuroprotective, with few animals developing trigeminal ganglion infection and none developing brain infection. These results suggest that a single vaccine formulation, containing *gC2*, *gD2*, or *gE2* nucleoside-modified mRNA, or perhaps modified to include additional immunogens, may function as a pan-herpes simplex vaccine by protecting against genital and non-genital infections caused by HSV-1 and HSV-2.

## Data Availability

The original contributions presented in this study are included in the article. Further inquiries can be directed to the corresponding author.
